# Characteristics of replicated single-nucleotide polymorphism genotypes from COGA: Affymetrix and Center for Inherited Disease Research

**DOI:** 10.1186/1471-2156-6-S1-S154

**Published:** 2005-12-30

**Authors:** Nathan L Tintle, Kwangmi Ahn, Nancy Role Mendell, Derek Gordon, Stephen J Finch

**Affiliations:** 1 Department of Applied Mathematics and Statistics, Stony Brook University, Stony Brook, New York, 11794 USA; 2 Laboratory of Statistical Genetics, Rockefeller University, New York, New York, 10021 USA

## Abstract

Genetic Analysis Workshop 14 provided re-genotyped single-nucleotide polymorphism (SNP) data. Specifically, both Center for Inherited Disease Research (CIDR) and Affymetrix genotyped the same 11,560 SNPs from the Affymetrix GeneChip Mapping 10K Array marker set on the same 184 individuals from the Collaborative Study on the Genetics of Alcoholism database. While the inconsistency rate between CIDR and Affymetrix (two different genotypes for the same subject) was low (0.2%), the non-replication rate (two different genotypes for the same subject or one identified genotype and one missing genotype) was substantial (9.5%). The missing data could be from no-call regions, which is inconsistent with recent recommendations about the use of no-call regions in association tests. In addition, no-call regions would suggest that the actual inconsistency rate is higher than reported. A high inconsistency rate has significant impact on power in related hypothesis tests. In addition, the data are consistent with assumptions made in a recently proposed likelihood ratio test of association for re-genotyped data.

## Background

Reclassification (for this application to single-nucleotide polymorphism (SNP) genotyping, reclassification will be called re-genotyping) has been proposed as a real-time quality control measure to learn about the consistency of classifications [1-3]. Many researchers re-genotype a fraction (for Genetic Analysis Workshop 14 (GAW14) the re-genotype fraction was either 5% or 10%) of the sample as a way to confirm that the genotyping is valid and consistent. For GAW14 the re-genotyped data inconsistency rate was computed as number of inconsistents/total classifications. Typically, if this number is low enough (i.e., the data are relatively consistent) then the data are deemed valid, and analysis proceeds.

It has been shown by Tintle [4] that, with some assumptions, re-genotyping data can be used to estimate error rates, which in turn can be used to estimate true genotype distribution parameters. Subsequently, error rates can be used during the sample design phase to adjust power and sample size calculations (see Gordon et al. [5]). Tintle [4] also shows how error rate estimates can be incorporated into a likelihood ratio test of association. Power in an association test can be improved through the use of the re-genotyped information, and when re-genotyping costs are low enough, it can be cost effective to re-genotype. This work is based on two assumptions: 1) heterozygote-to-homozygote error rates are equal to homozygote-to-heterozygote error rates and 2) the homozygote-to-homozygote error rates are zero. However, this work is merely a theoretical presentation based on simulation. The GAW14 Collaborative Study on the Genetics of Alcoholism (COGA) data provides real data to examine the validity of the assumptions.

Current technology classifies SNP genotypes using a continuous scale, with mutually exclusive intervals representing different genotypes [6,7]. A no-call region is an interval, typically between two genotype intervals, for which no genotype is assigned [8]. That is, if a particular data value falls into that region, the genotype is assigned a missing value. When systematic missing data is present, it is possible that a no-call region was used to identify genotypes. Kang et al. [9] demonstrate that using a no-call region in genotyping tests of association does not improve the power. Essentially Kang et al. shows that using the no-call region gives a more accurate but smaller sample. However, this is not better than using the data without the no-call region: a larger, but less accurate, sample.

## Methods

### Definitions

#### Genotype

One of three mutually exclusive and exhaustive categories of identification. The three categories are denoted AA, AB, and BB. In some cases in which genotype data is unavailable the genotype is denoted "missing."

#### Consistency

Two genotypes for a particular SNP and subject exist and are the same (e.g., Center for Inherited Disease Research (CIDR) says BB and Affymetrix also says BB for SNP 2 on subject 10000012).

#### Inconsistency

Two genotypes for a particular SNP and subject exist and are different (e.g., CIDR says AB and Affymetrix says AA for SNP 4766 on subject 10001513).

#### Replication

Two genotypes for a particular SNP and subject exist and are the same or are both missing (e.g., CIDR says BB and Affymetrix also says BB for SNP 2 on subject 10000012, or both CIDR and Affymetrix say missing for SNP 32 on subject 10000899).

#### Non-replication

Two genotypes for a particular SNP and subject exist and are different or one of the two genotypes is missing (e.g., CIDR says AB and Affymetrix says AA for SNP 4766 on subject 10001513 or Affymetrix says AB and CIDR is missing for SNP 45 on subject 10000452).

### Data handling issues

This paper examines raw data from the CIDR replication of the Affymetrix chip for 184 individuals. The Affymetrix chip used was the Affymetrix GeneChip Mapping 10K Array marker set, providing a complete genome scan of 11,560 SNPs. There were 440 SNPs dropped from the analysis because they were not included in the final map information. Also, 5 of the 184 subjects were dropped. Two of the five were dropped because they had the same CIDR ID number, while the other three subjects had information on only 11,119 SNPs and no information to indicate which SNP variable was not on file. Thus, the analysis was conducted on 179 individuals and 11,120 SNPs, with each SNP genotyped by both CIDR and Affymetrix.

## Results

### Consistency of results

For the consistency analysis, missing data values are ignored. Table 1 shows a cross-classification of genotyping results from CIDR and Affymetrix. Homozygote-to-homozygote inconsistencies (AA to BB or BB to AA) occur in 0.00011% of the classifications (*n *= 2 of the 1,770,056 total number of classifications excluding categories with missing data). The four other inconsistent categories are of roughly the same magnitude (counts of 695, 785, 656, and 748). The three consistently identified categories are also of roughly the same magnitude. The inconsistency rate is 0.2% (*n *= 2,886 is the sum of the six categories of inconsistents out of 1,770,056).

### Replication of results

For the replication analysis, missing data values are included. We note that missing data values are about half as likely to occur in either the AA or BB category as in the AB category (see Tables 2 and 3 for the probabilities). The non-replication rate is 9.5%, (*n *= 189,003 is the sum of all off main diagonal values in Table 1 out of the total number classifications: 1,999,480). The missing-missing rate is 1.7% (*n *= 34,307).

## Discussion

With no gold standard available, inconsistency is the best available estimate of true error rates. However, it requires the assumption that errors occur independently for Affymetrix and CIDR. With this assumption, results are consistent with the two assumptions of Tintle [4]. First, homozygote-to-homozygote inconsistencies are extremely infrequent (0.00011%), suggesting that homozygote-to-homozygote errors are rare. Further, the other four inconsistent cells are roughly equal, and the distributions of the called genotypes (AA, AB, BB) from both Affymetrix and CIDR are approximately uniform. These facts suggest that the heterozygote-to-homozygote and homozygote-to-heterozygote error rates are roughly equal.

There also appears to be a pattern in the missing data rates. Specifically, 2*P(*AA is missing*) = *P*(*AB is missing*) = 2*P(*BB is missing*). Kang et al. [9] identifies a procedure that would create such a distribution of missing values. The situation described by Kang et al. requires 1) an underlying univariate continuous measurement, 2) the conditional distribution of the measurement be normal for each group (genotype), 3) the distribution groups have equal variance, 4) the mean of group AB is half-way between the means of groups AA and BB (e.g., AA~N(-d, σ^2^), AB~N(0, σ^2^), and BB~N(d, σ^2^), where d is some constant greater than 0), and 5) there are two no-call regions of length 2r centered halfway between the homozygote and heterozygote means (e.g., , where r is some constant greater than 0). Under these conditions, when data values are distributed equally among categories (i.e., there are the same number of AA, AB, and BB), the observed missing data rates will follow a 1:2:1 distribution. Because the row and column marginals of the called genotypes are roughly equivalent, and the data follows a 1:2:1 distribution, it is possible that no-call regions were used while genotyping.

If missing data were occurring independently across all SNPs and individuals, the Missing – Missing Rate would equal (1/3)*(P(AA is missing)^2^+P(AB is missing)^2^+P(BB is missing)^2^) = (1/2)*P(AB is missing)^2^, where P(genotype i is missing) is the conditional probability of missing data after a single classification (see Tables 2 and 3 for the observed rates). The predicted missing – missing rate under the independence assumption is significantly less than the observed rate. However, we also note that the relative main diagonal symmetry in table 1 suggests independence when SNPs are identified.

## Conclusion

While the inconsistency rate was quite small, the large non-replication rate (due to missing data) is of interest. It appears that data are missing systematically. As was described above, a 1:2:1 pattern of missing data follows a no-call region genotyping procedure proposed by Kang et al. [9]. If no-call regions were used, careful attention should to be paid to Kang's work because it shows that no-call regions are not cost-effective for testing association. No-call regions contribute to the low inconsistency rates. If the no-call regions were removed and cut-points were used instead, the inconsistency rate would likely increase.

The use of inconsistency rates to estimate error [4] has implications for the power of association tests. Gordon et al. [5] show that for tests of association, the implications of large error rates on power is substantial. However, further inquiry is necessary to establish the true cause of the missing data.

In addition to the missing data described above, there was also a substantial amount of data that was missing for both Affymetrix and CIDR – much more than would be expected under independence. Further investigation is necessary to establish the reason for this missing data.

Because the data are consistent with the assumptions proposed by Tintle [4], his proposed likelihood ratio test of association for re-genotyped data is a good candidate for use on the data. Further work is necessary to confirm the theoretical result that the use of the re-genotyped data will improve the association test result.

## Abbreviations

CIDR: Center for Inherited Disease Research

COGA: Collaborative Study on the Genetics of Alcoholism

GAW14: Genetic Analysis Workshop 14

SNP: Single-nucleotide polymorphism

## Authors' contributions

NLT conducted some analyses, participated in the development of research goals, drafted and revised the manuscript, and provided the theoretical framework. KA arranged the database, conducted the majority of analyses, and participated in the development of research goals. NRM, DG, and SJF participated in the development of research goals, gave extensive feedback on findings, and provided expertise in the field of SNP genotyping. SJF also supervised the data analysis. All authors read and approved the final manuscript.

## Figures and Tables

**Table 1:** Cross-classification of regenotyping results summed over all SNPs and individuals

	CIDR				
		
	AA	AB	BB	Missing	Total
Affymetrix					
AA	593,662	785	1	25,843	620,291
AB	695	583,922	656	46,896	632,169
BB	1	748	589,586	26,547	616,882
Missing	20,996	45,178	20,657	34,307	121,138
Total	615,354	630,633	610,900	133,593	1,990,480^a^

^a^11,120 SNPs × 179 individuals = 1,990,480 total classifications

**Table 2:** Conditional probabilities of Affymetrix missing data

CIDR genotype	Probability Affymetrix is missing
AA	20,996/615,354 = 3.41%
AB	45,178/630,633 = 7.16%
BB	20,657/610,900 = 3.38%

**Table 3:** Conditional probabilities of CIDR missing data

Affymetrix genotype	Probability CIDR is missing
AA	25,843/620,291 = 4.17%
AB	46,896/632,169 = 7.42%
BB	26,547/616,882 = 4.30%
